# Antibiotic resistance in the pathogenic foodborne bacteria isolated from raw kebab and hamburger: phenotypic and genotypic study

**DOI:** 10.1186/s12866-021-02326-8

**Published:** 2021-10-07

**Authors:** Maryam Rajaei, Mir-Hassan Moosavy, Sahar Nouri Gharajalar, Seyed Amin Khatibi

**Affiliations:** 1 Department of Food Hygiene and Aquatic, Faculty of Veterinary Medicine, University of Tabriz, Tabriz, Iran; 2 Department of Pathobiology, Faculty of Veterinary Medicine, University of Tabriz, Tabriz, Iran

**Keywords:** Foodborne pathogens, Kebab, Hamburger, Antibiogram, Antibiotic-resistance genes

## Abstract

**Background:**

In recent years, interest in the consumption of ready-to-eat (RTE) food products has been increased in many countries. However, RTE products particularly those prepared by meat may be potential vehicles of antibiotic-resistance foodborne pathogens. Considering kebab and hamburger are the most popular RTE meat products in Iran, this study aimed to investigate the prevalence and antimicrobial resistance of common foodborne pathogens (*Escherichia coli*, *Salmonella* spp., *Staphylococcus aureus*, and *Listeria monocytogenes*) in raw kebab and hamburger samples collected from fast-food centers and restaurants. Therefore, total bacterial count (TBC), as well as the prevalence rates and antibiogram patterns of foodborne pathogens in the samples were investigated. Also, the presence of antibiotic-resistance genes (*bla*_SHV_, *bla*_TEM,_ *bla*_Z_, and *mec*A) was studied in the isolates by PCR.

**Results:**

The mean value of TBC in raw kebab and hamburger samples was 6.72 ± 0.68 log CFU/g and 6.64 ± 0.66 log CFU/g, respectively. *E. coli* had the highest prevalence rate among the investigated pathogenic bacteria in kebab (70%) and hamburger samples (48%). *Salmonella* spp., *L. monocytogenes,* and *S. aureus* were also recovered from 58, 50, and 36% of kebab samples, respectively. The contamination of hamburger samples was detected to *S. aureus* (22%), *L. monocytogenes* (22%), and *Salmonella* spp. (10%). In the antimicrobial susceptibility tests, all isolates exhibited high rates of antibiotic resistance, particularly against amoxicillin, penicillin, and cefalexin (79.66–100%). The *bla*_TEM_ was the most common resistant gene in the isolates of *E. coli* (52.54%) and *Salmonella* spp. (44.11%). Fourteen isolates (23.72%) of *E. coli* and 10 isolates (29.41%) of *Salmonella* spp. were positive for *bla*_SHV_. Also, 16 isolates (55.17%) of *S. aureus* and 10 isolates (27.27%) of *L. monocytogenes* were positive for *mec*A gene.

**Conclusions:**

The findings of this study showed that raw kebab and hamburger are potential carriers of antibiotic-resistance pathogenic bacteria, which can be a serious threat to public health.

**Supplementary Information:**

The online version contains supplementary material available at 10.1186/s12866-021-02326-8.

## Background

Antibiotics are commonly used for the treatment of infectious diseases in humans and animals [1–3]. In recent years, the excessive and uncontrolled use of antibiotics in veterinary medicine has become a major area of concern for human health. One of the main consequences of antibiotic residues in foods of animal origin is the proliferation of antibiotic-resistant bacteria. The presence of antibiotic-resistant pathogenic bacteria in foods may lead to hard-to-treat foodborne infections in humans. They can also transfer the resistance genes to other microorganisms through the food chain [2–5].

Multidrug resistance has increased globally that is considered a public health threat. Several previous investigations revealed the emergence of multidrug-resistant bacterial pathogens from different origins especially, in the food chain that increases the need for proper application of the antimicrobial agents in both veterinary and health sectors [6–13].

Antibiotic resistance limits the selection of therapeutic agents and increases the potential for treatment failures and adverse clinical complications. The presence of extended-spectrum antibiotic resistance genes (such as extended-spectrum beta-lactamases) in bacteria has been a major concern for public health [14].

Nowadays, due to the problems caused by industrialization, the interest of people toward the use of ready-to-eat (RTE) products has been increased [15]. However, these products may be prepared at low hygienic conditions by food handlers [16–18]. Therefore, RTE foods, particularly those prepared by meat, have been considered as potential vehicles of bacterial foodborne pathogens [17]. Meat is known as a rich source of high-quality animal proteins, vitamins B, and most of the trace minerals which are essential in human nutrition [19]. Due to the nutrient contents of meat, it provides an ideal medium for the growth of microorganisms [20].

Food products of animal origin such as meat and meat products are the main vehicles for the transmission of food-borne zoonotic bacterial pathogens. *Escherichia coli* (*E. coli*)*, Staphylococcus aureus* (*S. aureus*), *Salmonella* spp. and *Listeria monocytogenes* (*L. monocytogenes*) have been known as the major zoonotic bacterial pathogens which are associated with many cases of foodborne illness and death in humans following the consumption of contaminated food in the world [21–26].

Meat and meat products may be an important vehicle for the dissemination of antibiotic-resistant pathogenic bacteria [1–3, 27]. Several studies in recent years have been reported the presence of antibiotic-resistance bacteria in meat and meat products [27–31]. Therefore, monitoring the prevalence of antibiotic-resistance microorganisms not only is necessary to provide enough knowledge about the magnitude of this problem but also help governmental authorities to evaluate the effectiveness of control measures [2–4].

Due to the lack of proper surveillance systems in developing countries such as Iran, there are little scientific data available regarding the prevalence of foodborne pathogens in RTE meat products in these countries. Moreover, the antimicrobial resistance of foodborne pathogens in kebab and hamburgers were rarely investigated. To date, there is little knowledge about the relationship that may exist between antibiotic resistance phenotypes and resistance genes in pathogenic organisms isolated from RTE meat products.

Kebab and hamburger are the most commonly used RTE meat products in Iran. Therefore, the current study was aimed to evaluate the prevalence, and the phenotypic and genotypic profile of antibiotic-resistance *E. coli*, *Salmonella* spp., *S. aureus*, and *L. monocytogenes* isolated from raw kebab and hamburger samples which was collected from fast-food centers and restaurants in the northwest of Iran. Also, the presence of antibiotic resistance genes of *bla*_TEM_, *bla*_SHV_, *bla*_Z_, and *mec*A were investigated in the isolates.

## Results

### Total bacterial count (TBC)

The mean total colony forming unit per gram (CFU/g) of raw kebab (*n* = 50) and hamburger samples (*n* = 50) was 6.72 ± 0.68 log CFU/g and 6.64 ± 0.66 log CFU/g, respectively. However, the difference between the mean values of TBC in kebab and hamburger samples was not statistically significant (*p* > 0.05).

### Prevalence of different foodborne pathogens

*E. coli* had the highest prevalence rate (70%) in kebab samples between the investigated pathogenic bacteria. *Salmonella* spp., *L. monocytogenes,* and *S. aureus* were found in 58, 50, and 36% of kebab samples, respectively. Also, high rates of contamination to *E. coli* (48%), *S. aureus* (22%), *L. monocytogenes* (22%), and *Salmonella* spp. (10%) were detected in hamburger samples (Table 1).

**Table 1:** Prevalence of different foodborne pathogens in raw kebab and hamburger samples

Bacteria	Kebab (***n*** = 50)	Hamburger (***n*** = 50)
	No. of positive samples	No. of positive samples
***S. aureus***	18 (36%)	11 (22%)
***E. coli***	35 (70%)	24 (48%)
***Salmonella*** **spp.**	29 (58%)	5 (10%)
***L. monocytogenes***	25 (50%)	11 (22%)

### Antibiotic susceptibility patterns of the retrieved bacterial pathogens

**Fig. 1:**
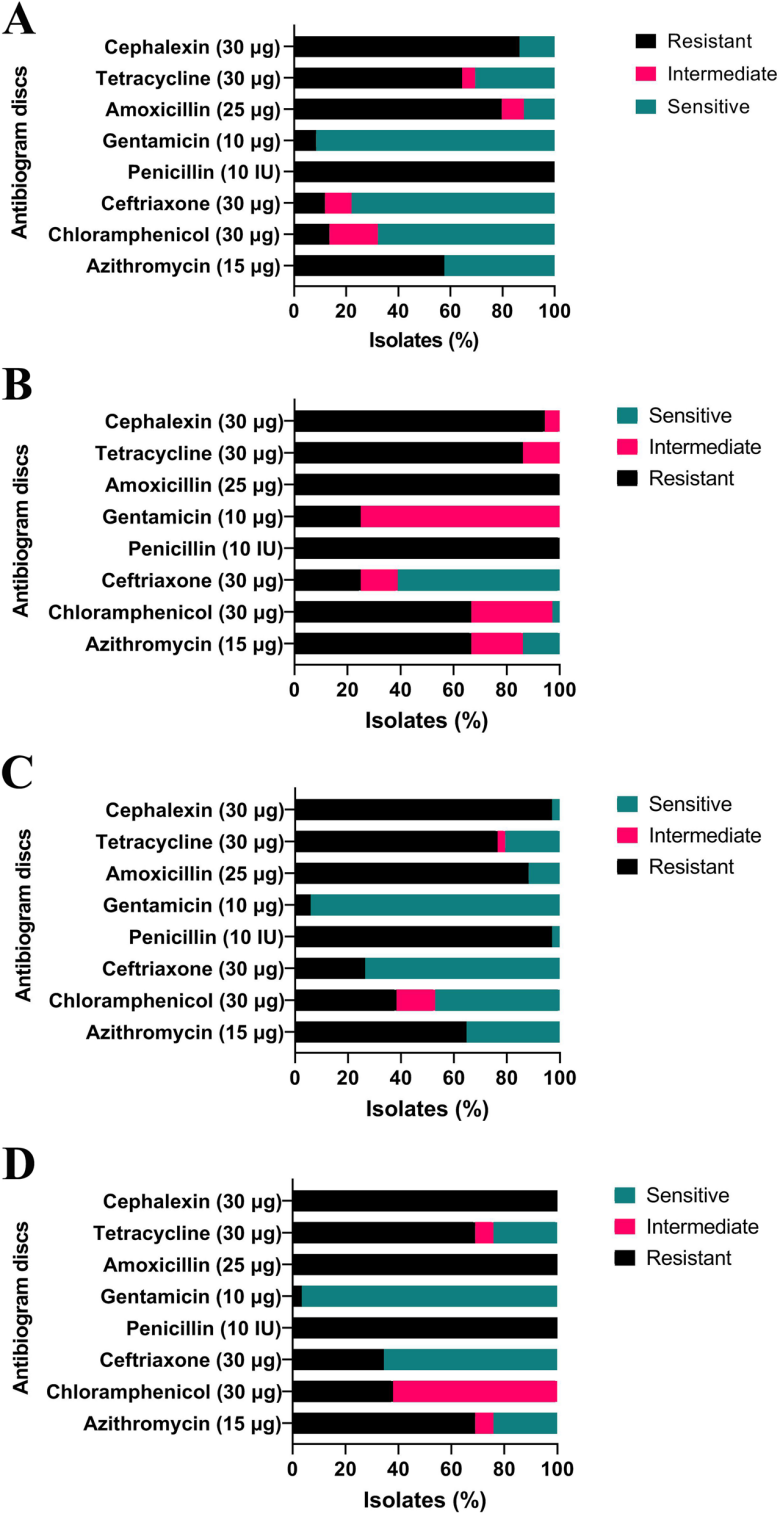
Antibiotic susceptibility pattern of *E. coli* (**A**), *L. monocytogenes* (**B**), *Salmonella* spp. (**C**) and *S. aureus* isolates (**D**) to the evaluated antibiotics

The resistance pattern of *E. coli* to the antibiotics studied is shown in Table 2 and Fig. 1A. The highest antibiotic resistance of *E. coli* isolated from kebab and hamburger samples was to penicillin (100%) followed by cephalexin (86.27%) and amoxicillin (80.00%), respectively. The highest antibiotic susceptibility of the isolates was observed to gentamicin (91.11%), ceftriaxone (78.00%), and chloramphenicol (67.34%), respectively. Resistance to ≥2 antimicrobials was found in all *E. coli* isolates. Three isolates of *E. coli* from hamburgers were resistant to all tested antibiotics (Table 6).

**Table 2:** Antibiotic resistance profile of *Escherichia coli* isolates from raw kebab and hamburger samples

Antibiotic class	Specific antibiotic tested	Concentration	Interpretive categories and zone diameter breakpoints (nearest whole mm)^a^			No. of isolates/Total isolates		
			R^b^	I	S	R	I	S
*Macrolides*	*Azithromycin*	*15 μg*	≤ 12	–	≥ 13	34/59	–	25/59
*Cephalosporins*	*Ceftriaxone*	*30 μg*	≤ 19	20–22	≥ 23	7/59	6/59	46/59
	*Cephalexin*	*30 μg*	≤ 14	–	≥ 15	51/59	–	8/59
*Penicillins*	*Penicillin*	*10 IU*	≤ 14	–	≥ 15	59/59	–	0/59
	*Amoxicillin*	*25 μg*	≤ 13	14–16	≥ 17	47/59	5/59	7/59
*Aminoglycosides*	*Gentamicin*	*10 μg*	≤ 12	13–14	≥ 15	5/59	0/59	54/59
*Tetracyclines*	*Tetracycline*	*30 μg*	≤ 11	12–14	≥ 15	38/59	3/59	18/59
*Phenicols*	*Chloramphenicol*	*30 μg*	≤ 12	13–17	≥ 18	8/59	11/59	40/59

^a^ From CLSI [32]

^b^ *R* resistant, *I* intermediate, *S* susceptible

*L. monocytogenes* isolates had the highest resistance against penicillin (100%), amoxicillin (100%), and cephalexin (93.75%) (Table 3) (Fig. 1B). Multidrug resistance to 6 antibiotics was observed in 42.66% of *L. monocytogenes* isolates including 48.00% of kebab isolates and 27.27% of hamburger isolates (Table 6).

**Table 3:** Antibiotic resistance profile of *Listeria monocytogenes* isolates from raw kebab and hamburger samples

Antibiotic class	Antibiotics	Concentration	Interpretive categories and zone diameter breakpoints (nearest whole mm)^a^			No. of isolates/Total isolates		
			R^b^	I	S	R	I	S
*Macrolides*	*Azithromycin*	*15 μg*	< 17	17–21	≥ 22	24/36	7/36	5/36
*Cephalosporins*	*Ceftriaxone*	*30 μg*	< 15	15–20	≥ 21	9/36	5/36	22/36
	*Cephalexin*	*30 μg*	< 12	12–17	≥ 18	34/36	2/36	0/36
*Penicillins*	*Penicillin*	*10 IU*	< 8	8–28	≥ 29	36/36	0/36	0/36
	*Amoxicillin*	*25 μg*	< 14	14–24	≥ 25	36/36	0/36	0/36
*Aminoglycosides*	*Gentamicin*	*10 μg*	< 18	18–20	≥ 21	9/36	27/36	0/36
*Tetracyclines*	*Tetracycline*	*30 μg*	< 22	22–24	≥ 25	31/36	5/36	0/36
*Phenicols*	*Chloramphenicol*	*30 μg*	< 18	18–20	≥ 21	24/36	11/36	1/36

^a^ From CLSI [33], Hansen et al. [34], CA-SFM [35]and Soussy et al. [36]

^b^ *R* resistant, *I* intermediate, *S* susceptible

The highest antibiotic resistance of *Salmonella* spp. isolates were detected to penicillin (97.5%) followed by cefalexin (96.77%) and amoxicillin (88.46%), respectively. The isolates were highly sensitive to gentamicin (94.11%) (Table 4) (Fig. 1C). Multi-drug resistance to more than 4 antibiotics was found in 91.17% of *Salmonella* spp. isolates. Two isolates of *Salmonella* spp. (6.89%) from kebab samples were resistant to all tested antibiotics (Table 6).

**Table 4:** Antibiotic resistance profile of *Salmonella* spp. isolates from raw kebab and hamburger samples

Antibiotic class	Specific antibiotic tested	Concentration	Interpretive categories and zone diameter breakpoints (nearest whole mm)^a^			No. of isolates/Total isolates		
			R^b^	I	S	R	I	S
*Macrolides*	*Azithromycin*	*15 μg*	≤ 12	–	≥ 13	22/34	–	12/34
*Cephalosporins*	*Ceftriaxone*	*30 μg*	≤ 19	20–22	≥ 23	9/34	0/34	25/34
	*Cephalexin*	*30 μg*	≤ 14	–	≥ 15	33/34	–	1/34
*Penicillins*	*Penicillin*	*10 IU*	≤ 14	–	≥ 15	33/34	–	1/34
	*Amoxicillin*	*25 μg*	≤ 13	14–16	≥ 17	30/34	0/34	4/34
*Aminoglycosides*	*Gentamicin*	*10 μg*	≤ 12	13–14	≥ 15	2/34	0/34	32/34
*Tetracyclines*	*Tetracycline*	*30 μg*	≤ 11	12–14	≥ 15	26/34	1/34	7/34
*Phenicols*	*Chloramphenicol*	*30 μg*	≤ 12	13–17	≥ 18	13/34	5/34	16/34

^a^From CLSI [32]

^b^ *R* resistant, *I* intermediate, *S* susceptible

The highest antibiotic resistance of *S. aureus* isolates was observed against penicillin, amoxicillin, and cephalexin. All of the isolates (100%) were resistant to the above antibiotics. The sensitivity of isolates was mostly determined to gentamicin (95.45%). Resistance to gentamicin was found only in one isolate (Table 5) (Fig. 1D). Multi-drug resistance to 7 antibiotics was found in 4 (22.22%) isolates from kebab and 2 (18.18%) isolates from hamburger. Overall, 86.20% of isolates were resistant to ≥4 antibiotics, concurrently (Table 6).

**Table 5:** Antibiotic resistance profile of *Staphylococcus aureus* isolates from raw kebab and hamburger samples

Antibiotic class	Specific antibiotic tested	Concentration	Interpretive categories and zone diameter breakpoints (nearest whole mm)^a^			No. of isolates/Total isolates		
			R^b^	I	S	R	I	S
*Macrolides*	*Azithromycin*	*15 μg*	≤ 13	14–17	≥ 18	20/29	2/29	7/29
*Cephalosporins*	*Ceftriaxone*	*30 μg*	≤ 13	14–20	≥ 21	10/29	0/29	19/29
	*Cephalexin*	*30 μg*	≤ 21	–	≥ 22	29/29	–	0/29
*Penicillins*	*Penicillin*	*10 IU*	≤ 28	–	≥ 29	29/29	–	0/29
	*Amoxicillin*	*25 μg*	≤ 28	–	≥ 29	29/29	–	0/29
*Aminoglycosides*	*Gentamicin*	*10 μg*	≤ 12	13–14	≥ 15	1/29	0/29	28/29
*Tetracyclines*	*Tetracycline*	*30 μg*	≤ 14	15–18	≥ 19	20/29	2/29	7/29
*Phenicols*	*Chloramphenicol*	*30 μg*	≤ 12	13–17	≥ 18	11/29	18/29	0/29

^a^From CLSI [32], CA-SFM [35]

^b^ *R* resistant, *I* intermediate, *S* susceptible

**Table 6:** Prevalence of multi-drug resistance in the selected foodborne pathogens isolated from raw kebab and hamburger samples

Food borne pathogens	No. of antibiotics	Overall ***n*** = 59 (%)	Kebab ***n*** = 35 (%)	Hamburger ***n*** = 24 (%)
***Escherichia coli***	1	0 (0.00)	0 (0.00)	0 (0.00)
	2	3 (5.08)	2 (5.71)	1 (4.16)
	3	16 (27.11)	11 (31.42)	5 (20.83)
	4	14 (23.72)	12 (34.28)	2 (8.33)
	5	7 (11.86)	7 (20.00)	0 (0.00)
	6	9 (15.25)	3 (8.57)	6 (25.00)
	7	7 (11.86)	0 (0.00)	7 (29.16)
	8	3 (5.08)	0 (0.00)	3 (12.50)
		**Overall** ***n*** **= 36 (%)**	**Kebab** ***n*** **= 25 (%)**	**Hamburger** ***n*** **= 11 (%)**
***Listeria monocytogenes***	1	0 (0.00)	0 (0.00)	0 (0.00)
	2	1 (2.77)	0 (0.00)	1 (9.09)
	3	4 (11.11)	3 (12.00)	1 (9.09)
	4	2 (5.55)	1 (4.00)	1 (9.09)
	5	6 (16.66)	5 (20.00)	1 (9.09)
	6	15 (42.66)	12 (48.00)	3 (27.27)
	7	5 (13.88)	3 (12.00)	2 (18.18)
	8	3 (8.33)	1 (4.00)	2 (18.18)
		**Overall** ***n*** **= 34 (%)**	**Kebab** ***n*** **= 29 (%)**	**Hamburger** ***n*** **= 5 (%)**
***Salmonella*** **spp.**	1	1 (2.94)	1 (3.44)	0 (0.00)
	2	1 (2.94)	1 (3.44)	0 (0.00)
	3	1 (2.94)	1 (3.44)	0 (0.00)
	4	9 (26.47)	7 (24.13)	2 (40.00)
	5	8 (23.52)	5 (17.24)	3 (60.00)
	6	10 (29.41)	10 (34.48)	0 (0.00)
	7	2 (5.88)	2 (6.89)	0 (0.00)
	8	2 (5.88)	2 (6.89)	0 (0.00)
	**No. of antibiotics**	**Overall** ***n*** **= 29 (%)**	**Kebab** ***n*** **= 18 (%)**	**Hamburger** ***n*** **= 11 (%)**
***Staphylococcus aureus***	1	0 (0.00)	0 (0.00)	0 (0.00)
	2	0 (0.00)	0 (0.00)	0 (0.00)
	3	4 (13.79)	1 (5.55)	3 (27.27)
	4	7 (24.13)	5 (27.77)	2 (18.18)
	5	4 (13.79)	3 (16.66)	1 (9.09)
	6	8 (27.58)	5 (6.25)	3 (27.27)
	7	6 (20.68)	4 (22.22)	2 (18.18)
	8	0 (0.00)	0 (0.00)	0 (0.00)

### Distribution of the antibiotic-resistance genes among the isolated bacterial pathogens

The prevalence rates of resistance genes in the isolates are presented in Table 7 based on the results of PCR test. The *bla*_TEM_ was the most common resistant gene in the isolates of *E. coli* (52.54%) and *Salmonella* spp. (44.11%). Fourteen isolates (23.72%) of *E. coli* and 10 isolates (29.41%) of *Salmonella* spp. were positive for *bla*_SHV_. Also, 55.17% of *S. aureus* isolates harbored the* mec*A gene. The *bla*_Z_ was present in two isolates (18.18%) of *S. aureus* from hamburger samples. Also, this gene was detected in three isolates (16.66%) from kebab samples. The *mec*A was observed in 10 isolates (27.27%) of *L. monocytogenes*.

**Table 7:** Prevalence of antibiotic-resistance genes in the selected foodborne bacterial isolated from kebab and hamburger samples

Pathogenic bacteria		No. of positive samples for target genes			
		***bla***_**SHV**_	***bla***_**Z**_	***bla***_**TEM**_	***mec***A
***E. coli***	Overall *n* = 59	14 (23.72%)	- ^a^	31 (52.54%)	–
	Kebab *n* = 35	7 (20.00%)	–	18 (51.42%)	–
	Hamburger *n* = 24	7 (29.16%)	–	13 (54.16%)	–
***Salmonella*** **spp.**	Overall *n* = 34	10 (29.41%)	–	15 (44.11%)	–
	Kebab *n* = 29	8 (27.58%)	–	12 (41.37%)	–
	Hamburger *n* = 5	2 (40.00%)	–	3 (60.00%)	–
***L. monocytogenes***	Overall *n* = 36	–	–	–	10 (27.27%)
	Kebab *n* = 25	–	–	–	8 (32.00%)
	Hamburger *n* = 11	–	–	–	2 (18.18%)
***S. aureus***	Overall *n* = 29	–	5 (17.24%)	–	16 (55.17%)
	Kebab *n* = 18	–	3 (16.66%)	–	10 (55.55%)
	Hamburger *n* = 11	–	2 (18.18%)	–	6 (54.54%)

^a^Not detected

The electrophoresis pattern of the PCR products of the resistance genes in the bacteria is shown in Figures S1, S2, S3 and S4.

## Discussion

In the present study, the contamination of raw hamburger and kebab to the selected pathogenic bacteria was investigated. Moreover, the antibiotic resistance pattern of the isolates and the presence of the resistance genes were studied. The results of this study exhibited the overall hygienic status of restaurants and fast food centers. Most of the raw kebabs and hamburgers collected from these locations did not have the proper bacteriological quality and high prevalence rates of contamination were observed in the samples to the selected pathogenic bacteria. According to the surveillance report for foodborne disease outbreaks in the United States during 2009–2015, among outbreaks reporting a single location of preparation, restaurants are the most commonly reported locations (2880 outbreaks [61%]), followed by catering or banquet facilities (636 [14%]) and private homes (561 [12%]). Restaurants with sit-down dining (2239 [48%]) and fast-food restaurants (369 [8%]) were the most commonly reported types of restaurants [37]. Many foodborne illnesses may occur by the secondary contamination of food and improper implementation of hygienic principles [38]. The results of the present study showed that raw kebabs and hamburgers prepared in restaurants and fast food centers could be a potential risk factor for public health.

Since microbial contamination in large numbers may cause rapid alterations in the organoleptic properties of the meat products; TBC is used as a common criterion to predict the shelf life of these products. Also, TBC is an expression of the hygienic quality level of foodstuffs [4]. According to European Union standards, the microbiological limit for TBC in meat preparations is 6.7 log CFU/g [39]. Also, based on the GMP guidelines, it has been recommended that the TBC level for raw meat preparations should not exceed 5 (maximum 7) log CFU/g [40, 41]. In the present study, the mean value for TBC in raw kebab samples was higher than 6.7 log CFU/g. Also, the mean levels of TBC in all samples exceed 5 log CFU/g. The high levels of TBC in this study are in agreement with the results of previous studies [4, 42, 43]. The high levels of microbial contamination in meat products may occur as the result of high contamination levels of raw materials and inappropriate processing conditions. Besides the microbial contamination of meat, the used spices, and other additives, the hygienic conditions of the processing environment, equipment, and handlers have significant effects on the TBC levels of meat products [4].

Since *E. coli* is known as a fecal indicator of RTE products [44], contamination of hamburger and kebab samples to this bacterium may cause foodborne disease in consumers.

In comparison with the results of the present study, lower contamination levels to *E. coli* have been reported in hamburger samples in Portugal (20%) [17] and beef samples in Saudi Arabia (22.22%) [45] and Ethiopia (6%) [46]. However, other authors reported the rates of 88.0% in beef samples in Ghana [1] and 100% of Beef preparations (meatballs, minced meat, hamburgers, white sausages, and red sausages) in the northwest of Spain [4]. Regarding the results of the present study, high levels of contamination to *E. coli* in RTE meat products may be due to the high contamination of the raw materials or preparation of products in an unhygienic condition.

In the present study, all hemolytic isolates and all isolates that were positive for *stx*1, *stx*2, or *eae* genes were considered as potential pathogenic *E. coli*. In kebab samples, 6 potential pathogenic *E. coli* isolates were detected (prevalence 12.0%). Of these 6 isolates, four isolates were categorized as Shiga toxin-producing strain (STEC) (8.0%). In hamburger samples, 4 potential pathogenic *E. coli* could be identified (prevalence 8.0%); of these, one was classified as STEC (2.0%).

*E. coli* is one of the most important pathogenic bacteria in nosocomial infections. It is known as the most important cause of endemic and epidemic diarrhea in the world [24, 25, 47–49]. Although the human gut flora is composed of a large number of bacterial species, *E. coli* exhibits greater antibiotic resistance than other Enterobacteriaceae, and this problem has been increased in both developed and developing countries [50]. The antibiotic-resistance strains of *E. coli* can be transmitted between animals and humans through the food chain. They can also transfer their resistance genes to other pathogens [51].

In agreement with the results of the present study, Hemeg [45] found that *E. coli* isolates from beef meat samples were highly resistant to penicillin (100%) and amoxicillin-clavulanic acid (100%). All amoxicillin-clavulanic acid-resistant *E. coli* isolates were positive for the *bla*_TEM_ gene. Moreover, *bla*_SHV_ resistant gene was detected in 60.52% of isolate. Also, Alegría et al. [52] in a study about the presence of β-lactam-resistant *E. coli* in food samples, reported that 80 and 20% of isolates carried *bla*_TEM_ and *bla*_SHV_ genes, respectively. Similar to the results of the present study, Ramadan et al. [53] reported that the highest resistance of *E. coli* isolates from different resources including retail ground beef in Mansoura, Egypt was to ampicillin. A high rate of resistance to amoxicillin and penicillin in the present study may be associated with the role of *bla*_SHV_ and *bla*_TEM_ genes in the antibiotic resistance of *E. coli*. It has been reported that TEM and SHV extended-spectrum beta-lactamases are responsible for the resistance against ampicillin, carbenicillin, cephalothin, and extended-spectrum cephalosporins [54].

The *bla*_TEM_ gene was the most common resistance gene in *E. coli* isolates in this study. However, only 77.41% of the resistance isolates to both amoxicillin and penicillin harbored this resistance gene. Also, 57.14% of isolates positive for *bla*_SHV_ showed resistance to cephalexin, penicillin, and amoxicillin in phenotypic experiments. It has been reported that the *E. coli* strains that are phenotypically positive for ESBL production but genotypically negative for ESBL genes can also be regarded as ESBL producers. Because most of the phenotypically-positive isolates do not harbor all existing resistance genes [55].

*Listeria monocytogenes* is a foodborne pathogen that is responsible for a disease in humans and animals, called listeriosis. In healthy people, it can cause febrile gastroenteritis with influenza-like symptoms. However, an acute disease may occur with the symptoms of encephalitis, meningitis, and septicemia in newborns, pregnant women, and immunocompromised and elderly people with a high rate of mortality (20–30%). This organism can be transmitted to human through the consumption of meat, dairy, poultry, fish, and vegetable products [26, 28, 56]. The results of the present study showed that 22.0% of the raw hamburger samples were contaminated with *L. monocytogenes*. Similar prevalence rates have been reported in retail raw meat products in other countries. In a study conducted in Italy, *L. monocytogenes* was detected in 23.6% of raw meat samples [57]. However, lower prevalence rates have been reported by some authors. In Turkey, Doğruer et al. [28] found that 1.25, 7.5, and 5% of meat pieces, minced meat, and hamburger samples were positive for *L. monocytogenes*. Ozbey et al. [58] in a study on raw hamburger meatballs and chicken burgers obtained from different fast food and markets in eastern Turkey found that 5.7% of hamburger meatballs were positive for *L. monocytogenes*. However, this organism was isolated from 13.9% of chicken burgers. The prevalence of *L. monocytogenes* in RTE meat products collected from seven regions in China was detected only 0.64% [59].

In agreement with the results of the present study, Nemati et al. [60] in a study on retail RTE meat products in Gorgan province, Iran, reported that most isolates of *L. monocytogenes* were resistant to penicillin and ampicillin. Wan et al. [61] found that the resistant rates of *L. monocytogenes* strains in China to tetracycline and chloramphenicol were 50.25 and 0.49%, respectively. However, in the present study, higher rates of resistance were observed to tetracycline (86.11%) and chloramphenicol (66.66%) in *L. monocytogenes* isolates.

Different results have been reported about the presence of the *mec*A gene in *L. monocytogenes* isolates by other authors. In a study on antibiotic resistance profiles of *L. monocytogenes* isolated from chicken meat in Fukuoka (Japan) in 2017, 94.7% of isolates were positive for *mecA* [56]*.* However, Wang et al. [61] reported that all experimental strains of foodborne listeria were negative for this gene. The *mec*A gene encodes a penicillin-binding protein [56], which is probably involved in the high resistance of all *L. monocytogenes* isolates to penicillin in this study. However, only 25.00% of them harbored the *mec*A gene. This result indicates that other genes may also be involved in the resistance of isolates to penicillin.

*Salmonella* spp. is known as the most important zoonotic foodborne pathogens after the campylobacter. Chicken, turkey, pork, beef, and other meats as well as eggs are the most important sources of this pathogen [62]. The intact tissues of healthy animals are sterile. However, after the slaughtering of these animals, the surface of the meat can be contaminated with these bacteria by the animal skin, the contents of the lumen, or handling. Therefore, observation of the hygienic principles is necessary during the slaughter of livestock and the preparation of the meat product [63, 64]. *Salmonella* spp. are sensitive organisms to heating processes. So, enough heat can kill this bacterium during food cooking. Meat can be a potential source of salmonellosis for the consumers, if it has primary contamination or is secondarily contaminated during the preparation or the use of additives, and it is not given enough heat during the cooking process [63]. Since kebab and hamburger can be prepared by hands from red meat, the cooking heat is not possibly enough to eliminate the primary or secondary contamination.

Taghizadeh et al. [65] reported that the prevalence of *Salmonella* spp. in hamburger samples in Mazandaran province (Iran) was 48.18%. In another study in Mexico, *Salmonella* spp. was determined in ground beef (56.7%). However, in the present study, *Salmonella* spp. was detected in 10% of hamburger samples. The probable reason for lower contamination levels of hamburger samples than kebabs could be related to the fact that raw hamburgers are usually kept in freezers in restaurants and fast-food centers, while raw kebab samples were stored in the refrigerator.

Antibiotics are necessary to treat Salmonella-induced enteritis particularly when there is a risk of acute infection (e.g. for infants, elderly and immunocompromised individuals) [66].

Nowadays, due to increased antibiotic resistance of Salmonella strains, fluoroquinolones and third-generation cephalosporins are usually used in the treatment of Salmonella infections. Other Antibiotics such as ampicillin, chloramphenicol, and cotrimoxazole, which were once the most widely used in the treatment of these infections, are less commonly used in recent years [67, 68]. In the present study, *Salmonella* spp. isolates had high resistance rates to penicillin, cephalexin, amoxicillin, and tetracycline. Also, six isolates (17.64%) with multidrug resistance to penicillin, amoxicillin, and cephalexin in the phenotypic study, harbored both *bla*_TEM_ and *bla*_SHV_ genes.

Similar to the findings of this study, high rates of antibiotic resistance in *Salmonella* spp. isolates to penicillins, tetracyclines, and cephalosporins have been reported by other authors. Altaf Hussain et al. [20] found that Salmonella isolates from retail raw beef in Karachi city, Pakistan were highly resistant to ampicillin (90.5%), amoxicillin (81.1%), and tetracycline (76%). Fortuna et al. [69] found that the *Salmonella* spp. isolated from beef and chicken hamburgers, were resistant to cefotaxime (88.89%), ampicillin (71.11%), cephalothin (68.89%), ceftriaxone (53.33%), cefoxitin (48.89%), and ceftazidime (42.22%). In the other study about chicken and beef meat samples as well as internal organs in northern Egypt, the resistance of *Salmonella enterica* serovars to ampicillin, cefotaxime, cefpodoxime, and tetracycline were 86.7, 80.0, 60.0, and 40.0%, respectively [70]. The results of previous studies were in agreement with the findings of the present work about the presence of *bla*_TEM_ and *bla*_SHV_ in *Salmonella* spp. isolates. Moawad et al. [70] detected *bla*_TEM_ in 73.3% of Salmonella isolates from chicken and beef meat samples in northern Egypt. The presence of *bla*_TEM_ was also reported in 17% of Salmonella isolates from retail meats in Alberta, Canada, while *bla*_SHV_ was not detected in the isolates [71]. In a study on retail raw beef in Karachi city, Pakistan, it was reported that *bla*_TEM_ were the dominant resistant genes in SalmonellaEnteritidis (*S. *Enteritidis) (24.0%) and SalmonellaTyphimurium (*S. *Typhimurium) (14.5%) followed by *Salmonella* Pullorum (*S*. Pullorum) (2%) whereas *bla*_SHV_ was the least detected β-lactamase gene in isolates of *S. *Enteritidis (2.6%), *S. *Typhimurium (5%) and *S*. Pullorum (2%) [20].

Due to the wide use of raw meat in the preparation of hamburgers and kebab as well as the increasing interest in the use of RTE foods, there is a possibility for the contamination of these products with *S. aureus* resulting in foodborne intoxication in the consumers. Contamination of RTE foods to *S. aureus* may be due to a variety of reasons, including a lack of proper hygiene during the preparation [72]. Aycicek et al. [73] reported that food handlers play a major role in *S. aureus* contamination of ready-to-eat products.

The results of previous studies about the contamination of hamburger and meat products with *S. aureus* are in agreement with the finding of the present study. The prevalence rates of *S. aureus* in hamburger samples have been reported to be 25% in Tehran (Iran) [74] and 20% in Poland [75]. In a meta-analysis study in Ethiopia, the prevalence rates of *Staphylococcus* spp. in beef and other animal meats were 21 and 22%, respectively [46]. Arafa et al. [76] in a study in Cairo (Egypt) found that 30% of minced beef meat samples and 10% of beef burger samples were positive for *S. aureus.*

Many authors have reported the high resistance level of *S. aureus* isolates to penicillins and tetracyclines that are in agreement with the results of the antimicrobial susceptibility test in the present study. Çetinkaya and Elal Mus [77] reported that the *S. aureus* isolates from different foodstuffs including raw meatballs in Turkey were resistant to penicillin (62.9%) and ampicillin (59.3%). Resistant of *S. aureus* isolates (57.14%) from the beef burger and beef minced meat to penicillin and methicillin has also been reported in Cairo, Egypt [76]. In a similar study on raw retail meat samples collected from Isfahan province, Iran, *S. aureus* isolates exhibited the highest resistance to tetracycline (79.16%), penicillin (72.91%), and doxycycline (41.66%) [78].

The *mec*A and *bla*_Z_ genes are common genes involved in antibiotic resistance of *S. aureus* strains. In the study of Shahraz et al. [74] and Chajęcka-Wierzchowska et al. [75] on hamburgers samples, the *mec*A gene was detected in 100% of the Methicillin-resistant *S. aureus* isolates. Arafa et al. [76] also reported the presence of the *mecA* and *blaZ* gene in 85.7% of *S. aureus* isolates from minced beef meat and burger samples, while all of the isolates (100%) were positive for the *bla*_Z_ gene. In the study of Baghbaderani et al. [78], the *bla*_Z_ gene was detected in 58.33% of the isolates from raw retail meat samples. In the present study, these genes were detected in the *S. aureus* isolates from hamburger and kebab samples. Notably, all of the resistance isolates to penicillin, amoxicillin, and cephalexin in the phenotypic tests, harbored both mecA and *bla*_Z_ genes. Penicillin is usually used as the drug of choice for the treatment of infections caused by *S. aureus*. However, it has been recently reported that approximately 90% of human *S. aureus* are resistant to penicillin. The* blaZ* gene has been suggested as the main mechanism responsible for penicillin resistance in *Staphylococci*. Also, the *bla*Z gene can transfer between coagulase-negative *Staphylococci* (as the resistance gene reservoir) and *S. aureus.* Moreover, the production of penicillin-binding protein, PBP2a, encoded by *mec*A is proposed as the second primary mechanism for penicillin resistance particularly in the human isolates [79].

In this study, it was observed that the phenotypic resistance pattern was different from the presence of associated resistance genes in the isolates. For example, resistance to penicillins in the phenotypic study was more prevalent than the related resistance genes in the isolates. These results indicate that possession of a certain phenotypic resistance pattern does not always accurately correlate with a resistance gene. While the antibiotic resistance genes may be mutated or not expressed, other mechanisms of resistance such as multidrug efflux pumps, mutations in outer membrane porins, or other unknown resistance genes may be effective in the phenotypic resistance pattern [80, 81].

In the present study, high resistance levels and multidrug resistances against up to eight antibiotics were observed in the isolates, with a high proportion for β-lactams. Since beta-lactams are the most commonly used antibiotics in human and veterinary medicine, the emergence of β-lactam-resistant pathogenic bacteria can be a big challenge for public health [82].

## Conclusion

The findings of this study showed that kebab and hamburger, as the widely consumed RTE meat products, have a high prevalence of important foodborne pathogens showing multi-resistance to most commonly used antibiotics of therapeutic importance in human medicine. Although kebab and hamburger are usually not consumed in raw form, consumption of improperly cooked products and possible cross-contamination to other foodstuffs can pose a major potential risk to public health. The discrepancies between the phenotypic resistances and associated resistance genes in the isolates indicated that possession of a certain phenotypic resistance pattern may be related to other resistance mechanisms.

The present study highlights the urgent need for precise observation of hygienic principles by food handlers, appropriate authority supervision, and regulatory monitoring to ensure that safe RTE meat products are prepared for the consumers. To better understand the epidemiology of antibiotic resistance in foodborne pathogens, further studies should be focused on other RTE foods and their potential risk for transmission of multi-drug resistance pathogens. Also, it is suggested that in future studies, serotyping assays be performed on the isolates to identify the common serovars of pathogenic bacteria with antibiotic resistant in RTE meat products.

## Methods

### Sampling

A total of 100 samples of kebab and hamburger (50 samples, each) were collected from restaurants and fast food centers in Tabriz city, Iran from May 2018 to September 2019. The samples were transported immediately to the laboratory and kept under refrigeration (4 ± 1 °C). The study was conducted in the laboratory of Food microbiology in the Faculty of Veterinary Medicine, University of Tabriz.

### Total bacterial count

Briefly, 25 g of the kebab or hamburger samples were placed in a sterile Pulsifer bag containing 225 mL of 0.1% sterile peptone water [83]. The contents of the bags were homogenized for 2 min using the Pulsifier (Microgen Bioproducts, Surrey, UK). Decimal dilutions of homogenate samples were prepared in test tubes containing 9 ml of 0.1% peptone water. One (1) ml of each dilution was pour plated on plate count agar (Merck, Darmstadt, Germany). The plates were incubated at 37 °C for 48 h, and the colony counts were calculated [84].

### Isolation and identification of pathogenic bacteria

#### *Escherichia coli*

Isolation of *E. coli* was performed according to the FDA method [85] and Ombarak et al. [86]. Briefly, *E. coli* was identified in the samples using Lauryl tryptose (LST) broth (Merck, Germany) containing Durham tube. After incubation at 37 °C for 48 h, 1 ml of the bacterial suspension from positive tubes (determined by turbidity and gas production) was transferred to the brilliant green bile lactose broth (BGLB) (Merck, Germany) and incubated at 37 °C for 48 h. Aliquot of suspension (0.1 ml) from positive tubes were streaked on the Eosin Methylene Blue (EMB) agar plates and incubated at 37 °C for 24 h. The presumptive colonies (dark centered and flat colonies with metallic green sheen) were was picked and streaked on tryptic soy agar (TSA) (Merck, Germany), and incubated at 37 °C for 24 h. The colonies were cultured on the slants of nutrient agar at 37 °C for 16 h and used for biochemical analysis. The isolates were confirmed by Gram staining, growth on MacConkey Agar (Merck, Darmstadt, Germany), growth in brilliant green bile lactose broth (Merck, Darmstadt, Germany), and biochemical tests such as IMViC, oxidase, catalase, motility tests, sugar fermentation and nitrate reduction (Table S1) [1, 87]. Finally, the isolates of pathogenic *E. coli* were detected after hemolysis test on enterohemolysin agar (Oxoid, Germany) as well as PCR test for the presence of *stx*1 (primers forward: 5′-GTGGTTGCGAAGGAATTTACC-3′; reverse: 5′-ACTGATCCCTGCAACACGCTG-3′), *stx*2 (forward: 5′-ATCCTATTCCCGGGAGTTTACG; reverse: 5′-GCGTCATCGTATACACAGGAGC-3′) and *eae* (intimin) (forward: 5′-ATGCCCGGACCCGGCACAAG-3′; reverse: 5′-AAGAGTCTCGCCAGTATTCG-3′) genes [88].

#### *Listeria monocytogenes*

Isolation and identification of *L. monocytogenes* were carried out using the method of Food and Drug Administration (FDA) [83]. Briefly, 25 g of each sample was mixed with 225 ml of Listeria enrichment broth (Merck, Darmstadt, Germany). The cultures were incubated at 30 °C for 4 h for the enrichment. Then, Listeria-Selective Enrichment Supplement (Merck, Darmstadt, Germany) was added to the broth and incubated for 44 h. A loopful from the enrichment broth was streaked onto Palcam Listeria Selective agar (Merck, Darmstadt, Germany) and incubated for 48 h at 35 °C. The grey-green colonies with a black center and black halo were subjected to the confirmatory tests such as Gram staining, motility in SIM Medium and biochemical test (catalase, oxidase, hemolysis on blood agar, urea, nitrate reduction, MR-VP, CAMP test, esculin hydrolysis, and fermentation of glucose, mannitol, maltose, xylose, and rhamnose) (Table S1) [83].

#### *Salmonella* spp.

Isolation of *Salmonella* spp. was firstly performed by pre-enrichment of samples in lactose broth (Merck, Darmstadt, Germany) at 37 °C for 24 h. For selective enrichment, pre-enriched cultures were transferred into Selenite Cystine (SC) broth (Merck, Darmstadt, Germany) and *Tetrathionate* Brilliant Green bile (TBG) broth (Merck, Darmstadt, Germany), and incubated at 35 °C for 24 h. Then, these cultures were streaked onto Bismuth Sulphite agar (BSA) (Oxoid, Basingstoke, UK), Xylose Lysine Deoxycholate Agar (XLD) (Oxoid, Basingstoke, UK), and Hektoen Enteric agar (HEA) (Oxoid, Basingstoke, UK) as selective media and incubated at 35 °C for 48 h. Typical colonies were cultured on the slants of Tryptic soy agar (TSA) (Merck, Darmstadt, Germany) and subjected to biochemical tests using Lysine Iron agar (LIA) (Merck, Darmstadt, Germany), Triple Sugar Iron (TSI) agar (Merck, Darmstadt, Germany), Sulfide-Indole-Motility (SIM) medium (Merck, Darmstadt, Germany), and Christensen’s Urea agar (Merck, Darmstadt, Germany) (Table S1) [89].

#### *Staphylococcus aureus*

One (1) ml of sample solution was taken to Cooked-Meat broth (Merck, Germany) containing 10% NaCl and incubated at 37 °C for 24 h. Then, a loopful of culture was transferred onto Baird-Parker agar (Merck, Darmstadt, Germany) supplemented with egg yolk and tellurite emulsion (50 ml/l), and incubated at 37 °C for 24 h. Black shiny colonies surrounded by clear halo were confirmed by Gram staining and biochemical tests such as catalase activity, hemolytic activity on blood agar (Merck, Darmstadt, Germany), VP, urease, oxidation activity, fermentation of mannitol on Mannitol salt agar (Merck, Darmstadt, Germany), production of coagulase, and DNase test (Table S1) [74, 78].

### Antimicrobial susceptibility test

The antimicrobial susceptibility test was performed by the Kirby-Bauer disk diffusion method [90] according to the guidelines of clinical laboratory standards Institute (CLSI) [32]. Based on the interpretive categories and zone diameter breakpoints (nearest whole mm) given by CLSI, the inhibition zone diameter was measured and interpreted as resistant, intermediate, and susceptible. Duplicate isolates were excluded from the study based on isolation rank (time criterion). Using this criterion, the first isolate of a particular species isolated from a single sample during the study period was included in the analysis [91]. The isolates were tested against azithromycin (15 μg), chloramphenicol (30 μg), amoxicillin (25 μg), gentamicin (10 μg), penicillin (10 IU), ceftriaxone (30 μg), cephalexin (30 μg), and tetracycline (30 μg) (Patan-Teb Company, Iran). The selected antimicrobials were representative of the major classes of antimicrobial drugs commonly used in veterinary and human medicine. The isolates were inoculated in Trypticase Soy Broth (TSB) (Merck, Darmstadt, Germany) at 37 °C for 18 h. The turbidity of microbial suspension was adjusted to 0.5 McFarland standard using sterile TSB. The isolates were cultured separately on Müller–Hinton agar (Merck, Darmstadt, Germany). The antibiotic discs were placed on the agar (with intervals of 3 cm) and incubated at 37 °C for 24 h. The zones of growth inhibition were measured and the results were presented according to the guidelines of CLSI [32].

### PCR assays

#### DNA extraction

Firstly, *L. monocytogenes* and *S. aureus* isolates were cultured on the blood agar (Merck, Darmstadt, Germany). *Salmonella* spp. and *E. coli* were grown on MacConkey agar. Typical colonies were transferred to the nutrient broth and incubated at 37 °C for 24 h. The bacterial DNA was extracted by the boiling method. Briefly, bacteria grown in the broth were suspended in 300 μL of deionized water. The suspension was heated at 100 °C for 10 min in a water bath followed by cooling in an ice bath for 5–10 min. Then, it was centrifuged at 13000×*g* for 5 min. Finally, the supernatant containing the bacterial DNA was transferred to a sterile microtube and used as the DNA template. The templates were stored at − 20 °C until the next stages of PCR analysis [92].

#### PCR-based detection of the antibiotic-resistance genes among the isolated bacterial pathogens

The isolates were tested for antibiotic-resistance genes of *bla*_TEM_, *bla*_SHV_, *bla*_Z_, and *mec*A using the specific primers (Table 8). Firstly, 5.5 μL of deionized water was added to 3 μL of template DNA in a microtube. Then, 1 μL of each primer (Forward and Reverse), was added to the solution. Finally, 12.5 μL of RED-Extract-N-Amp master mix 2× (containing buffer, salts, dNTPs, Taq polymerase, REDTaq dye, and JumpStart Taq antibody) (Sigma-Aldrich, USA) was added. The mixture was then put in the thermocycler (MWG AC Biotech Thermal Cycler, USA). The PCR condition for *mec*A and *bla*_Z_ were as follows: initial denaturation at 94 °C for 2 min, 30 cycles of denaturation at 94 °C for 1 min, annealing at 55 °C for 1 min, the extension at 72 °C for 2 min and the final extension at 72 °C for 5 min. The PCR condition for *bla*_TEM_ and *bla*_SHV_ were as follows: initial denaturation at 94 °C for 5 min, 32 cycles of denaturation at 94 °C for 30 s, annealing at 54 °C for 30 s, the extension at 72 °C for 1 min and a final extension at 72 °C for 10 min.

**Table 8:** The PCR primers used in this study

Target gene	Primer sequence (5′ → 3′)	Amplicon size (bp)	Reference
*bla*Z	F: TGA CCA CTT TTA TCA GCA ACC R: GCC ATT TCA ACA CCT TCT TTC	700	[93, 94]
*mec*A	F: AAA ATC GAT GGT AAA GGT TGG C R: AGT TCT GCA GTA CCG GAT TTG C	532	[95–97]
*bla*_TEM_	F: ATC AGC AAT AAA CCA GC R: CCC CGA AGA ACG TTT TC	516	[98–100]
*bla*_SHV_	F: AGG ATT GAC TGC CTT TTT G R: ATT TGC TGA TTT CGC TCG	392	[101–103]

#### Electrophoresis of PCR products

PCR products were subject to electrophoresis using 1.5% agarose gel in 0.5X TBE buffer (0.1 M Tris, 0.1 M boric acid and 0.002 M NaEDTA). A 100-bp DNA ladder (Thermo Scientific, USA) was used as a molecular size standard. The gel was stained with 0.1% ethidium bromide, allowed to run at 75 V for 90 min. The amplicons were visualized under UV light using a Gel documentation system (Biorad, USA).

## Statistical analysis

All measurements were performed in triplicate. The total microbial counts were calculated as log CFU/g and presented as the mean ± standard deviation. The data were analyzed by the chi-square test and Fisher’s exact test using SPSS 16.0 software (SPSS Inc., Chicago, IL, USA). The results were considered to be statistically different at 95% confidence levels.

## Supplementary Information

**Additional file 1: Tables S1.** Results of biochemical tests from the selected foodborne pathogens isolated from kebab and hamburger samples.

**Additional file 2: Figure S1.** Agarose gel electrophoresis of PCR amplification products of *bla*_TEM_ (516 bp) and *bla*_SHV_ (392 bp) from *Escherichia coli* isolates, L: Ladder (100 bp); Lane 1: positive control for *bla*_TEM_; Lane 2: positive control for *bla*_SHV_; Lanes 5, 7, 9 and 10: positive samples for *bla*_TEM_; Lanes 5 and 9: positive samples for *bla*_SHV_.

**Additional file 3: Figure S2.** Agarose gel electrophoresis of PCR amplification products of *mec*A (532 bp) from *Listeria monocytogenes* isolates, L: Ladder (100 bp); Lanes 2, 3, 4 and 5: positive samples; Lane 6: positive control; Lane 7: negative control.

**Additional file 4: Figure S3.** Agarose gel electrophoresis of PCR amplification products of *bla*_SHV_ (392 bp) and *bla*_TEM_ (516 bp) genes from the isolates of *Salmonella* spp., L: Ladder (100 bp); Lanes 1: positive samples for *bla*_TEM_, Lanes 2 and 3: positive samples for *bla*_SHV_ *bla*_TEM_; Lane 6: positive control for *bla*_SHV_ *bla*_TEM_; Lane 7: negative control.

**Additional file 5: Figure S4.** Agarose gel electrophoresis of PCR amplification products of *mec*A (532 bp) and *bla*_Z_ (700 bp) from *Staphylococcus aureus* isolates, L: Ladder (100 bp); Lanes 2: positive sample for *mec*A and *bla*_Z_; Lanes 3 and 4: positive samples for *mec*A; Lanes 5: positive control for *mec*A and *bla*_Z_; Lane 6: negative control.

## Data Availability

The datasets generated and/or analysed during the current study are available in the Harvard Dataverse Repository, (10.7910/DVN/E0LCXA).

## Abbreviations

BSA: Bismuth sulphite agar
BGLB: Brilliant green bile lactose broth
BGA: Brilliant green phenol red agar
CAMP: Christie-Atkins-Munch-Peterson
CFU: Colony-forming unit
CLSI: Clinical laboratory standards Institute
DNase: Deoxyribonuclease
EMB: Eosin methylene blue
*E. coli*: *Escherichia coli*
ESBL: Extended-spectrum beta-lactamase
HEA: Hektoen enteric agar
LIA: Lysine iron agar
MR/VP: Methyl red and voges-proskauer
SC: Selenite cystine
SIM: Sulfide-indole-motility
*S. aureus*: *Staphylococcus aureus*
*S.* Enteritidis: S. Enteritidis
*S. *Typhimurium: SalmonellaTyphimurium
*S*. Pullorum: Salmonella Pullorum
*L. monocytogenes*: *Listeria monocytogenes*
PCR: Polymerase chain reaction
TBC: Total bacterial count
TBG: Tetrathionate brilliant green bile
TSA: Tryptic soy agar
RTE: Ready-to-eat
TSB: Trypticase soy broth
TSI: Triple sugar iron
XLD: Xylose lysine deoxycholate

## References

[1] Adzitey F (2020). Incidence and antimicrobial susceptibility of *Escherichia coli* isolated from beef (meat muscle, liver and kidney) samples in Wa abattoir, Ghana. Cogent Food Agric.

[2] Khatibi SA, Hamidi S, Siahi-Shadbad MR. Current trends in sample preparation by solid-phase extraction techniques for the determination of antibiotic residues in foodstuffs: a review. Crit Rev Food Sci Nutr. 2020:1–22 10.1080/10408398.2020.1798349. 10.1080/10408398.2020.1798349 32744053

[3] Khatibi SA, Hamidi S, Siahi-Shadbad MR. Application of liquid-liquid extraction for the determination of antibiotics in the foodstuff: recent trends and developments. Crit Rev Anal Chem. 2020:1–16 10.1080/10408347.2020.1798211. 10.1080/10408347.2020.1798211 32748637

[4] González-Gutiérrez M, García-Fernández C, Alonso-Calleja C, Capita R. Microbial load and antibiotic resistance in raw beef preparations from Northwest Spain. Food Sci Nutr. 2020;8(2):777–85. 10.1002/fsn3.1319. 10.1002/fsn3.1319 PMC7020325 32148787

[5] Javadi A, Khatibi SA (2017). Effect of commercial probiotic (Protexin®) on growth, survival and microbial quality of shrimp (*Litopenaeus vannamei*). Nutr Food Sci.

[6] Algammal A, Mabrok M (2019). Pathogenicity, genetic typing, and antibiotic sensitivity of *vibrio alginolyticus* isolated from *Oreochromis niloticus* and *Tilapia zillii*. Rev Med Vet.

[7] Algammal AM, El-Sayed ME, Youssef FM, Saad SA, Elhaig MM, Batiha GE, Hozzein WN, Ghobashy MOI (2020). Prevalence, the antibiogram and the frequency of virulence genes of the most predominant bacterial pathogens incriminated in calf pneumonia. AMB Express.

[8] Algammal AM, Enany ME, El-Tarabili RM, Ghobashy MOI, Helmy YA (2020). Prevalence, antimicrobial resistance profiles, virulence and enterotoxins-determinant genes of MRSA isolated from subclinical bovine mastitis in Egypt. Pathogens..

[9] Algammal AM, Hetta HF, Elkelish A, Alkhalifah DHH, Hozzein WN, Batiha GE-S, El Nahhas N, Mabrok MA (2020). Methicillin-resistant *Staphylococcus aureus* (MRSA): one health perspective approach to the bacterium epidemiology, virulence factors, antibiotic-resistance, and zoonotic impact. Infect Drug Resist..

[10] Algammal AM, Mabrok M, Sivaramasamy E, Youssef FM, Atwa MH, El-kholy AW, Hetta HF, Hozzein WN (2020). Emerging MDR-*Pseudomonas aeruginosa* in fish commonly harbor *opr*L and *tox*A virulence genes and *bla*TEM, *bla*CTX-M, and *tet*A antibiotic-resistance genes. Sci Rep.

[11] Algammal AM, Mohamed MF, Tawfiek BA, Hozzein WN, El Kazzaz WM, Mabrok M (2020). Molecular typing, antibiogram and PCR-RFLP based detection of *Aeromonas hydrophila* complex isolated from *Oreochromis niloticus*. Pathogens..

[12] Enany ME, Algammal AM, Nasef SA, Abo-Eillil SAM, Bin-Jumah M, Taha AE, Allam AA (2019). The occurrence of the multidrug resistance (MDR) and the prevalence of virulence genes and QACs resistance genes in *E. coli* isolated from environmental and avian sources. AMB Express.

[13] Abolghait SK, Fathi AG, Youssef FM, Algammal AM (2020). Methicillin-resistant *Staphylococcus aureus* (MRSA) isolated from chicken meat and giblets often produces staphylococcal enterotoxin B (SEB) in non-refrigerated raw chicken livers. Int J Food Microbiol.

[14] Novovic K, Mihajlovic S, Vasiljevic Z, Filipic B, Begovic J, Jovcic B (2015). Carbapenem-resistant *Acinetobacter baumannii* from Serbia: revision of *Car*O classification. PLoS One.

[15] Newell DG, Koopmans M, Verhoef L, Duizer E, Aidara-Kane A, Sprong H, Opsteegh M, Langelaar M, Threfall J, Scheutz F, der Giessen JV, Kruse H (2010). Food-borne diseases — the challenges of 20 years ago still persist while new ones continue to emerge. Int J Food Microbiol.

[16] Pereira PM, Vicente AF (2013). Meat nutritional composition and nutritive role in the human diet. Meat Sci.

[17] Campos J, Gil J, Mourão J, Peixe L, Antunes P (2015). Ready-to-eat street-vended food as a potential vehicle of bacterial pathogens and antimicrobial resistance: an exploratory study in Porto region, Portugal. Int J Food Microbiol.

[18] Gilani A, Razavilar V, Rokni N, Rahimi E (2017). *Vac*A and *cag*A genotypes status and antimicrobial resistance properties of *helicobacter pylori* strains isolated from meat products in Isfahan province, Iran. Iran J Vet Res.

[19] Obeid R, Heil SG, Verhoeven MMA, van den Heuvel EGHM, de Groot LCPGM, Eussen SJPM (2019). Vitamin B12 intake from animal foods, biomarkers, and health aspects. Front Nutr.

[20] Altaf Hussain M, Wang W, Sun C, Gu L, Liu Z, Yu T, Ahmad Y, Jiang Z, Hou J (2020). Molecular characterization of pathogenic *Salmonella* spp. from raw beef in Karachi, Pakistan. Antibiotics.

[21] Latha C, Anu CJ, Ajaykumar VJ, Sunil B (2017). Prevalence of *Listeria monocytogenes*, *Yersinia enterocolitica*, *Staphylococcus aureus*, and *Salmonella enterica* Typhimurium in meat and meat products using multiplex polymerase chain reaction. Vet World.

[22] Abebe E, Gugsa G, Ahmed M (2020). Review on major food-borne zoonotic bacterial pathogens. J Trop Med.

[23] Ebrahimi A, Moosavy M-H, Khatibi SA, Barabadi Z, Hajibemani A. A comparative study of the antibacterial properties of milk from different domestic animals. Int J Dairy Technol. 2021;74(2):425–30. 10.1111/1471-0307.12757.

[24] Khatibi SA, Misaghi A, Moosavy MH, Akhondzadeh Basti A, Mohamadian S, Khanjari A. Effect of nanoliposomes containing *Zataria multiflora* Boiss. essential oil on gene expression of Shiga toxin 2 in *Escherichia coli* O157:H7. J Appl Microbiol. 2018;124(2):389–97. 10.1111/jam.13641. 10.1111/jam.13641 29152837

[25] Khatibi SA, Misaghi A, Moosavy M-H, Basti AA, Koohi MK, Khosravi P, et al. Encapsulation of *Zataria multiflora* Bioss. essential oil into nanoliposomes and in vitro antibacterial activity against *Escherichia coli* O157:H7. J Food Process Preserv. 2017;41(3):e12955. 10.1111/jfpp.12955.

[26] Moosavy M-H, Esmaeili S, Mortazavian AM, Mostafavi E, Habibi-Asl B, Hosseini H, et al. Behaviour of *Listeria monocytogenes* in Lighvan cheese following artificial contamination during making, ripening and storage in different conditions. Int J Dairy Technol. 2017;70(3):365–71. 10.1111/1471-0307.12372.

[27] Ahmed AM, Shimamoto T. Molecular analysis of multidrug resistance in Shiga toxin-producing *Escherichia coli* O157:H7 isolated from meat and dairy products. Int J Food Microbiol. 2015;193:68–73. 10.1016/j.ijfoodmicro.2014.10.014. 10.1016/j.ijfoodmicro.2014.10.014 25462925

[28] Doğruer Y, Telli N, Telli E, Güner A (2015). Presence and antibiotic susceptibility of *Listeria monocytogenes* in retail meat and meat products. Int J Biol Res.

[29] Chajęcka-Wierzchowska W, Zadernowska A, Łaniewska-Trokenheim Ł (2016). Diversity of antibiotic resistance genes in Enterococcus strains isolated from ready-to-eat meat products. J Food Sci.

[30] Aguilar-Montes de Oca S, Talavera-Rojas M, Soriano-Vargas E, Barba-León J, Vázquez-Navarrete J, Acosta-Dibarrat J, Salgado-Miranda C (2018). Phenotypic and genotypic profile of clinical and animal multidrug-resistant *Salmonella enterica* isolates from Mexico. J Appl Microbiol.

[31] Jaja IF, Bhembe NL, Green E, Oguttu J, Muchenje V (2019). Molecular characterisation of antibiotic-resistant *Salmonella enterica* isolates recovered from meat in South Africa. Acta Trop.

[32] CLSI. Performance standards for antimicrobial susceptibility testing. 28th ed. CLSI supplement M100.; Clinical and laboratory standards institute. 2018. available at: https://clsi.org/media/1930/m100ed28_sample.pdf. (accessed 04 May 2020).

[33] CLSI. Methods for antimicrobial dilution and disk susceptibility testing of infrequently isolated or fastidious bacteria. 3th ed. CLSI guideline M45; Clinical and Laboratory Standards Institute. 2015. available at: https://clsi.org/standards/products/microbiology/documents/m45/. (accessed 04 May 2020).

[34] Hansen JM, Gerner-Smidt P, Bruun B (2005). Antibiotic susceptibility of *Listeria monocytogenes* in Denmark 1958-2001. Apmis..

[35] CA-SFM. Comité de l'Antibiogramme de la Société Française de Microbiologie Report 2003. Int J Antimicrob Agents. 2003;21(4):364–91. 10.1016/S0924-8579(03)00021-9. 10.1016/s0924-8579(03)00021-9 12672587

[36] Soussy CJ, Cluzel R, Courvalin P (1994). Definition and determination of in vitro antibiotic susceptibility breakpoints for bacteria in France. The Comité de l'Antibiogramme de la Société Française de Microbiologie. Eur J Clin Microbiol Infect Dis.

[37] Dewey-Mattia D, Manikonda K, Hall AJ, Wise ME, Crowe SJ (2018). Surveillance for foodborne disease outbreaks - United States, 2009-2015. MMWR Morb Mortal Wkly Rep Surveill Summ.

[38] Hawkes C, Blouin C, Henson S, Drager N, Dubé L (2009). Trade, food, diet and health: perspectives and policy options.

[39] Communities, CotE (2005). Commission Regulation (EC) No. 2073/2005 of 15 November 2005 on microbiological criteria for foodstuffs. OJEU.

[40] ICMSF (2011). Microorganisms in foods 8. Use of data for assessing process control and product acceptance.

[41] IFST (1997). Development and use of microbiological criteria for foods. Food Sci Technol Today.

[42] Andritsos ND, Mataragas M, Mavrou E, Stamatiou A, Drosinos EH. The microbiological condition of minced pork prepared at retail stores in Athens, Greece. Meat Sci. 2012;91(4):486–9. 10.1016/j.meatsci.2012.02.036. 10.1016/j.meatsci.2012.02.036 22459497

[43] Siriken B (2004). The microbiological quality of ground beef. Rev Med Vet.

[44] Cho JI, Cheung CY, Lee SM, Ko SI, Kim KH, Hwang IS, Kim SH, Cho SY, Lim CJ, Lee KH, Kim KS, Ha SD (2011). Assessment of microbial contamination levels of street-vended foods in Korea. J Food Saf.

[45] Hemeg HA. Molecular characterization of antibiotic resistant *Escherichia coli* isolates recovered from food samples and outpatient clinics, KSA. Saudi J Biol Sci. 2018;25(5):928–31. 10.1016/j.sjbs.2018.01.016. 10.1016/j.sjbs.2018.01.016 PMC6087806 30108443

[46] Zelalem A, Sisay M, Vipham JL, Abegaz K, Kebede A, Terefe Y (2019). The prevalence and antimicrobial resistance profiles of bacterial isolates from meat and meat products in Ethiopia: a systematic review and meta-analysis. Int J Food Contam..

[47] Sunabe T, Honma Y (1998). Relationship between O-serogroup and presence of pathogenic factor genes in *Escherichia coli*. Microbiol Immunol.

[48] Seas C, Alarcon M, Aragon JC, Beneit S, Quiñonez M, Guerra H, Gotuzzo E (2000). Surveillance of bacterial pathogens associated with acute diarrhea in Lima, Peru. Int J Infect Dis.

[49] Koo HJ, Woo GJ (2011). Distribution and transferability of tetracycline resistance determinants in *Escherichia coli* isolated from meat and meat products. Int J Food Microbiol.

[50] Osterblad M, Hakanen A, Manninen R, Leistevuo T, Peltonen R, Meurman O, Huovinen P, Kotilainen P (2000). A between-species comparison of antimicrobial resistance in enterobacteria in fecal flora. Antimicrob Agents Chemother.

[51] Van den Bogaard AE, Stobberingh EE (2000). Epidemiology of resistance to antibiotics. Links between animals and humans. Int J Antimicrob Agents.

[52] Alegría Á, Arias Temprano M, Fernández-Natal I, Rodríguez-Calleja J, García-López ML, Santos J (2020). Molecular diversity of ESBL-producing *Escherichia coli* from foods of animal origin and human patients. Int J Environ Res Public Health.

[53] Ramadan H, Jackson C, Hiott L, Samir M, Awad A, Woodley T (2020). Antimicrobial resistance, genetic diversity and multilocus sequence typing of *Escherichia coli* from humans, retail chicken and ground beef in Egypt. Pathogens..

[54] Helfand MS, Bonomo RA (2005). Current challenges in antimicrobial chemotherapy: the impact of extended-spectrum beta-lactamases and metallo-beta-lactamases on the treatment of resistant gram-negative pathogens. COPHAR..

[55] Schmid A, Hörmansdorfer S, Messelhäusser U, Käsbohrer A, Sauter-Louis C, Mansfeld R (2013). Prevalence of extended-spectrum β-lactamase-producing *Escherichia coli* on Bavarian dairy and beef cattle farms. Appl Environ Microbiol.

[56] Maung AT, Mohammadi TN, Nakashima S, Liu P, Masuda Y, Honjoh K-I, et al. Antimicrobial resistance profiles of *Listeria monocytogenes* isolated from chicken meat in Fukuoka, Japan. Int J Food Microbiol. 2019;304:49–57. 10.1016/j.ijfoodmicro.2019.05.016. 10.1016/j.ijfoodmicro.2019.05.016 31154111

[57] Pesavento G, Ducci B, Nieri D, Comodo N, Lo Nostro A (2010). Prevalence and antibiotic susceptibility of *Listeria* spp. isolated from raw meat and retail foods. Food Control.

[58] Ozbey G, Icyeroglu A, Muz A (2013). Prevalence of Listeria species in raw hamburger meatballs and chicken burgers in eastern Turkey. Afr J Microbiol Res.

[59] Yang S, Pei X, Wang G, Yan L, Hu J, Li Y, et al. Prevalence of food-borne pathogens in ready-to-eat meat products in seven different Chinese regions. Food Control. 2016;65:92–8. 10.1016/j.foodcont.2016.01.009.

[60] Nemati V, Khomeiri M, Sadeghi Mahoonak A, Moayedi A (2020). Prevalence and antibiotic susceptibility of *Listeria monocytogenes* isolated from retail ready-to-eat meat products in Gorgan, Iran. Nutr Food Sci Res.

[61] Wang TS, Wang Y, He CY, Ye ZX, Wang Y, Xu K, Yu H, Wang Y, Zhang L, Zeng Y, Xu X, Wang H, Chen W, Wang H, Xu XB (2013). Detection of drug susceptibility and resistant genes in selected food borne *Listeria monocytogens* in China. Dis Surveill.

[62] Roy P, Dhillon AS, Lauerman LH, Schaberg DM, Bandli D, Johnson S (2002). Results of salmonella isolation from poultry products, poultry, poultry environment, and other characteristics. Avian Dis.

[63] Gill CO, Jones T. The presence of Aeromonas, Listeria and Yersinia in carcass processing equipment at two pig slaughtering plants. Food Microbiol. 1995;12:135–41. 10.1016/S0740-0020(95)80089-1.

[64] Molla B, Alemayehu D, Abdela W (2002). Sources and distribution of Salmonella serotypes isolated from food animals, slaughterhouse personnel and retail meat products in Ethiopia: 1997-2002. EJHD..

[65] Taghizadeh M, Javadian B, Rafiei A, Taraghian A, Moosazadeh M (2019). Antimicrobial resistance and virulence of *Salmonella* spp. from foods in Mazandaran. Res Mol Med.

[66] Brooks GF, Butel JS, Morse SA, Brooks GF, Butel JS, Morse SA (2004). Cultivation of microorganisms. Jawetz, Melnick & Adelberg’s medical microbiology.

[67] Davis MA, Hancock DD, Besser TE, Rice DH, Gay JM, Gay C, Gearhart L, DiGiacomo R (1999). Changes in antimicrobial resistance among *Salmonella enterica* serovar Typhimurium isolates from humans and cattle in the northwestern United States, 1982-1997. Emerg Infect Dis.

[68] Su L-H, Wu T-L, Chia J-H, Chu C, Kuo A-J, Chiu C-H (2005). Increasing ceftriaxone resistance in Salmonella isolates from a university hospital in Taiwan. J Antimicrob Chemother.

[69] Fortuna J, Nascimento E, Franco R (2013). Antimicrobial resistance of *Salmonella* spp. strains isolated from hamburgers. Afr J Microbiol Res.

[70] Moawad AA, Hotzel H, Awad O, Tomaso H, Neubauer H, Hafez HM, El-Adawy H (2017). Occurrence of *Salmonella enterica* and *Escherichia coli* in raw chicken and beef meat in northern Egypt and dissemination of their antibiotic resistance markers. Gut Pathog.

[71] Aslam M, Checkley S, Avery B, Chalmers G, Bohaychuk V, Gensler G, et al. Phenotypic and genetic characterization of antimicrobial resistance in Salmonella serovars isolated from retail meats in Alberta, Canada. Food Microbiol. 2012;32(1):110–7. 10.1016/j.fm.2012.04.017. 10.1016/j.fm.2012.04.017 22850381

[72] Moosavy MH, Hassanzadeh P, Mohammadzadeh E, Mahmoudi R, Khatibi SA, Mardani K (2017). Antioxidant and antimicrobial activities of essential oil of Lemon (*Citrus limon*) peel in vitro and in a food model. ssu-jfqhc.

[73] Aycicek H, Cakiroglu S, Stevenson TH. Incidence of *Staphylococcus aureus i*n ready-to-eat meals from military cafeterias in Ankara, Turkey. Food Control. 2005;16(6):531–4. 10.1016/j.foodcont.2004.04.005.

[74] Shahraz F, Dadkhah H, Khaksar R, Mahmoudzadeh M, Hosseini H, Kamran M, Bourke P (2012). Analysis of antibiotic resistance patterns and detection of *mec*A gene in *Staphylococcus aureus* isolated from packaged hamburger. Meat Sci.

[75] Chajęcka-Wierzchowska W, Zadernowska A, Łaniewska-Trokenheim Ł (2017). *Staphylococcus aureus* from ready-to-eat food as a source of multiple antibiotic resistance genes. CBU Int Conf Proc.

[76] Arafa A, Ibrahim ES, Fouad E, Gaber ES (2016). Antibiotic resistance of *staphylococci* concerning strains included in food industry in Egypt. Int J Pharm Clin Res.

[77] Çetinkaya F, Elal Mus T (2012). Detection of antibiotic resistance in *Staphylococcus aureus* strains isolated from various foods. Uludağ Üniv Vet Fak Derg.

[78] Baghbaderani Z, Shakerian A, Rahimi E (2020). Phenotypic and genotypic assessment of antibiotic resistance of *Staphylococcus aureus* bacteria isolated from retail meat. Infect Drug Resist.

[79] Olsen JE, Christensen H, Aarestrup FM (2006). Diversity and evolution of *bla*Z from *Staphylococcus aureus* and coagulase-negative *staphylococci*. J Antimicrob Chemother.

[80] Davis MA, Besser TE, Orfe LH, Baker KNK, Lanier AS, Broschat SL, New D, Call DR (2011). Genotypic-phenotypic discrepancies between antibiotic resistance characteristics of *Escherichia coli* isolates from calves in management settings with high and low antibiotic use. Appl Environ Microbiol.

[81] Smith M, Do TN, Gibson JS, Jordan D, Cobbold RN, Trott DJ (2014). Comparison of antimicrobial resistance phenotypes and genotypes in enterotoxigenic *Escherichia coli* isolated from Australian and Vietnamese pigs. J Glob Antimicrob Resist.

[82] Ghazaei C (2018). Phenotypic and molecular detection of beta-lactamase enzyme produced by *Bacillus cereus* isolated from pasteurized and raw milk. J Med Bacteriol.

[83] Hitchins AD, Jinneman K, Chen Y. BAM chapter 10: detection of *Listeria monocytogenes* in foods and environmental samples, and enumeration of *Listeria monocytogenes* in foods: Food and Drug Administration; 2017. available at: https://www.fda.gov/food/laboratory-methods-food/bam-chapter-10-detection-listeria-monocytogenes-foods-and-environmental-samples-and-enumeration. (accessed 01 Apr 2021)

[84] Maharjan S, Rayamajhee B, Chhetri VS, Sherchan SP, Panta OP, Karki TB (2019). Microbial quality of poultry meat in an ISO 22000:2005 certified poultry processing plant of Kathmandu valley. Int J Food Contam.

[85] Feng P, Weagant SD, Grant MA, Burkhardt W, Shellfish M, Water B (2002). Bacteriological analytical manual: enumeration of *Escherichia coli* and the coliform bacteria.

[86] Ombarak R, Hinenoya A, Awasthi S, Iguchi A, Shima A, Elbagory A, Yamasaki S (2016). Prevalence and pathogenic potential of *Escherichia coli* isolates from raw milk and raw milk cheese in Egypt. Int J Food Microbiol.

[87] Barrow G, Feltham R (1993). Cowan and Steel's manual for the identification of medical bacteria.

[88] Mayrhofer S, Paulsen P, Smulders FJ, Hilbert F (2004). Antimicrobial resistance profile of five major food-borne pathogens isolated from beef, pork and poultry. Int J Food Microbiol.

[89] Andrews WH, Siliker J, Bailey JS, Labbe RG, Downes FPIK, FR (2001). Salmonella. Compendium of methods for the microbiological examination of foods.

[90] Bauer AW, Kirby WM, Sherris JC, Turck M (1966). Antibiotic susceptibility testing by a standardized single disk method. Am J Clin Pathol.

[91] Cornaglia G, Hryniewicz W, Jarlier V, Kahlmeter G, Mittermayer H, Stratchounski L, Baquero F (2004). European recommendations for antimicrobial resistance surveillance. Clin Microbiol Infect.

[92] Nayak R, Stewart TM, Nawaz MS (2005). PCR identification of *campylobacter coli* and *campylobacter jejuni* by partial sequencing of virulence genes. Mol Cell Probes.

[93] Kang MH, Chae MJ, Yoon JW, Kim SG, Lee SY, Yoo JH, Park HM (2014). Antibiotic resistance and molecular characterization of ophthalmic *Staphylococcus pseudintermedius* isolates from dogs. J Vet Sci.

[94] Meroni G, Soares Filipe JF, Drago L, Martino PA (2019). Investigation on antibiotic-resistance, biofilm formation and virulence factors in multi drug resistant and non multi drug resistant *Staphylococcus pseudintermedius*. Microorganisms..

[95] Shahmohammadi MR, Nahaei MR, Akbarzadeh A, Milani M (2016). Clinical test to detect mecA and antibiotic resistance in *Staphylococcus aureus*, based on novel biotechnological methods. Artif Cells Nanomed Biotechnol.

[96] Rocchetti TT, Martins KB, Martins PYF, Oliveira RA, Mondelli AL, Fortaleza C, Cunha M (2018). Detection of the *mec*A gene and identification of *Staphylococcus* directly from blood culture bottles by multiplex polymerase chain reaction. Braz J Infect Dis.

[97] Kim YH, Kim HS, Kim S, Kim M, Kwak HS (2020). Prevalence and characteristics of antimicrobial-resistant *Staphylococcus aureus* and methicillin-resistant *Staphylococcus aureus* from retail meat in Korea. Food Sci Anim Resour.

[98] Lyimo B, Buza J, Subbiah M, Smith W, Call DR (2016). Comparison of antibiotic resistant *Escherichia coli* obtained from drinking water sources in northern Tanzania: a cross-sectional study. BMC Microbiol.

[99] Abdel Aziz SA, Abdel-Latef GK, Shany SAS, Rouby SR (2018). Molecular detection of integron and antimicrobial resistance genes in multidrug resistant Salmonella isolated from poultry, calves and human in Beni-Suef governorate, Egypt. BJBAS.

[100] Eid S, Samir AH (2019). Extended-spectrum beta-lactamase and Class 1 integrons in multidrug-resistant *Escherichia coli* isolated from turkeys. Vet World..

[101] Tew LS, She LY, Chew CH (2016). Isolation, antimicrobial susceptibility profile and detection of *Sul*1, *bla*TEM, and *bla*SHV in amoxicillin-clavulanate-resistant bacteria isolated from retail sausages in Kampar, Malaysia. Jundishapur J Microbiol.

[102] Abrar S, Ain NU, Liaqat H, Hussain S, Rasheed F, Riaz S (2019). Distribution of *bla*CTX − M, *bla*TEM, *bla*SHV and *bla*OXA genes in extended-spectrum-β-lactamase-producing clinical isolates: a three-year multi-center study from Lahore, Pakistan. Antimicrob Resist Infect Control.

[103] Yukawa S, Uchida I, Tamura Y, Ohshima S, Hasegawa T (2019). Characterisation of antibiotic resistance of Salmonella isolated from dog treats in Japan. Epidemiol Infect.

