# Supplementary material for: Antibiotic resistance in the pathogenic foodborne bacteria isolated from raw kebab and hamburger: phenotypic and genotypic study
Source: BMC Microbiol. 2021 Oct 7;21:272. doi: 10.1186/s12866-021-02326-8 (PMC8495966; doi:10.1186/s12866-021-02326-8)
Supplement: Supplementary file 1 — Additional file 1: Tables S1. Results of biochemical tests from the selected foodborne pathogens isolated from kebab and hamburger samples. [file 12866_2021_2326_MOESM1_ESM.docx]

**Table S1.** Results of biochemical tests from the selected foodborne pathogens isolated from kebab and hamburger samples

|  | **Biochemical tests** | **Results** |
| --- | --- | --- |
| ***Staphylococcus aureus*** | Gram staining | + |
|  | Catalase | + |
|  | Hemolysis | + (Beta) |
|  | VP (Voges Proskauer) | - |
|  | Urease | + |
|  | Oxidase | - |
|  | Coagulase | + |
|  | DNase test | + |
|  | Fermentation of |  |
|  | Mannitol | + |
|  | Lactose | + |
|  | Trehalose | + |
|  | Sucrose | + |
|  | Glucose | + |
|  | Maltose | + |
|  | Xylose | - |
| ***Escherichia coli*** | Gram staining | - |
|  | Indole | + |
|  | Methyl red | + |
|  | VP | - |
|  | Citrate | - |
|  | Oxidase | - |
|  | Catalase | + |
|  | Motility | + |
|  | Nitrate reduction | + |
|  | Fermentation of |  |
|  | Lactose | + |
|  | Glucose | + |
|  | Xylose | + |
|  | Rhamnose | + |
|  | Trehalose | + |
| ***Listeria monocytogene*** | Gram staining | + |
|  | Motility | + |
|  | Oxidase | - |
|  | Hemolysis | + |
|  | Urease | - |
|  | Nitrate reduction | - |
|  | Methyl red | + |
|  | VP | + |
|  | CAMP test | + (Beta) |
|  | Esculin hydrolysis | + |
|  | Fermentation of |  |
|  | Glucose | + |
|  | Mannitol | - |
|  | Xylose | - |
|  | Rhamnose | + |
|  | Maltose | + |
| ***Salmonella* spp.** | Gram staining | - |
|  | Oxidase | - |
|  | Catalase | + |
|  | Lysine decarboxylase | + |
|  | TSI agar | Alkali/Acid |
|  | H_2_S | +/- |
|  | Motility | +/- |
|  | Urease | - |
|  | Methyl red | + |
|  | VP | - |
|  | Citrate | +/- |
|  | Indole | - |
|  | Fermentation of |  |
|  | Glucose | + |
|  | Lactose | - |
|  | Maltose | + |
|  | Mannitol | + |
|  | Dulcitol | +/- |
