# Supplementary figures and images for: Antibiotic resistance in the pathogenic foodborne bacteria isolated from raw kebab and hamburger: phenotypic and genotypic study
Source: BMC Microbiol. 2021 Oct 7;21:272. doi: 10.1186/s12866-021-02326-8 (PMC8495966; doi:10.1186/s12866-021-02326-8)

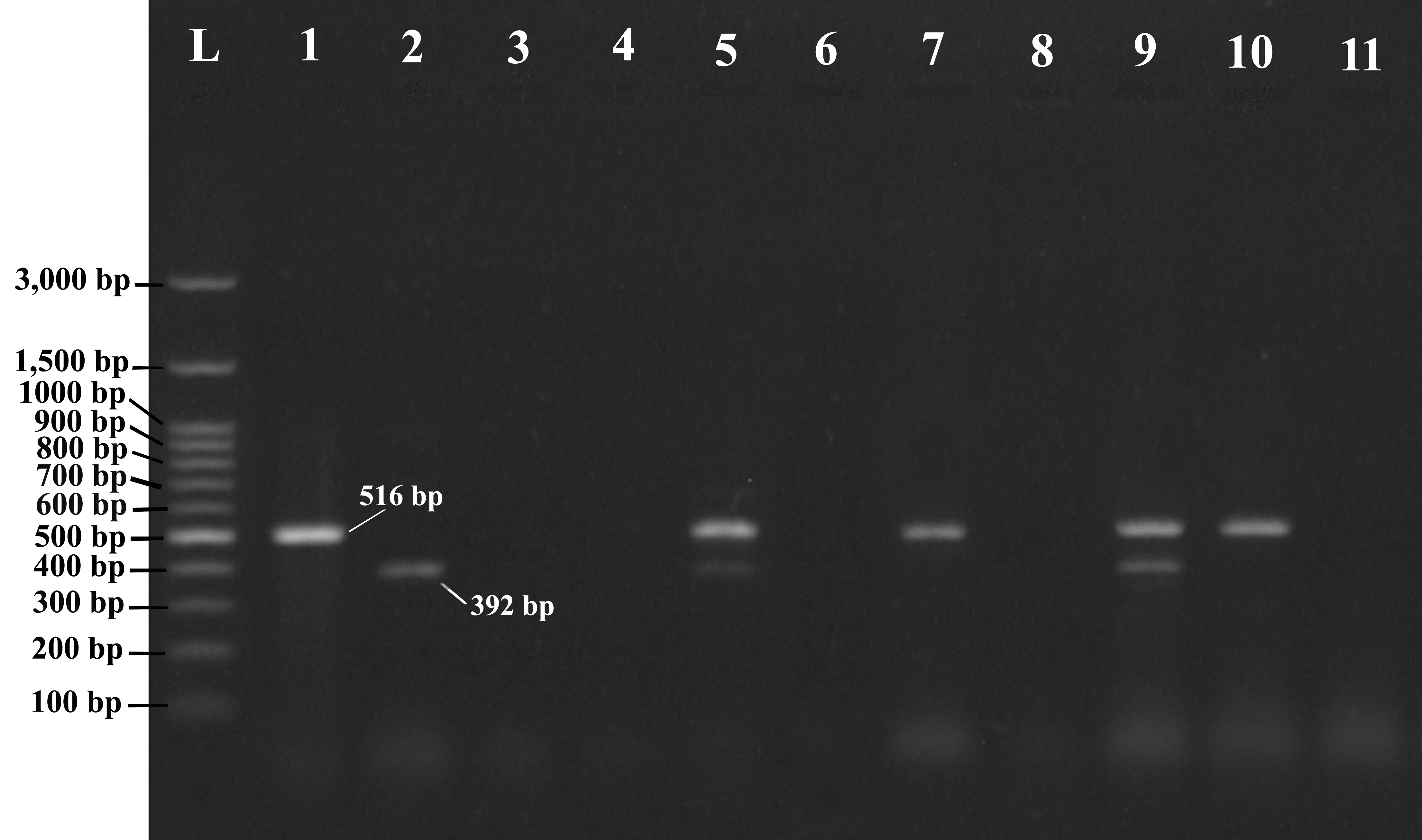

Supplement: Supplementary file 2 — Additional file 2: Figure S1. Agarose gel electrophoresis of PCR amplification products of blaTEM (516 bp) and blaSHV (392 bp) from Escherichia coli isolates, L: Ladder (100 bp); Lane 1: positive control for blaTEM; Lane 2: positive control for blaSHV; Lanes 5, 7, 9 and 10: positive samples for blaTEM; Lanes 5 and 9: positive samples for blaSHV. [file 12866_2021_2326_MOESM2_ESM.jpg]

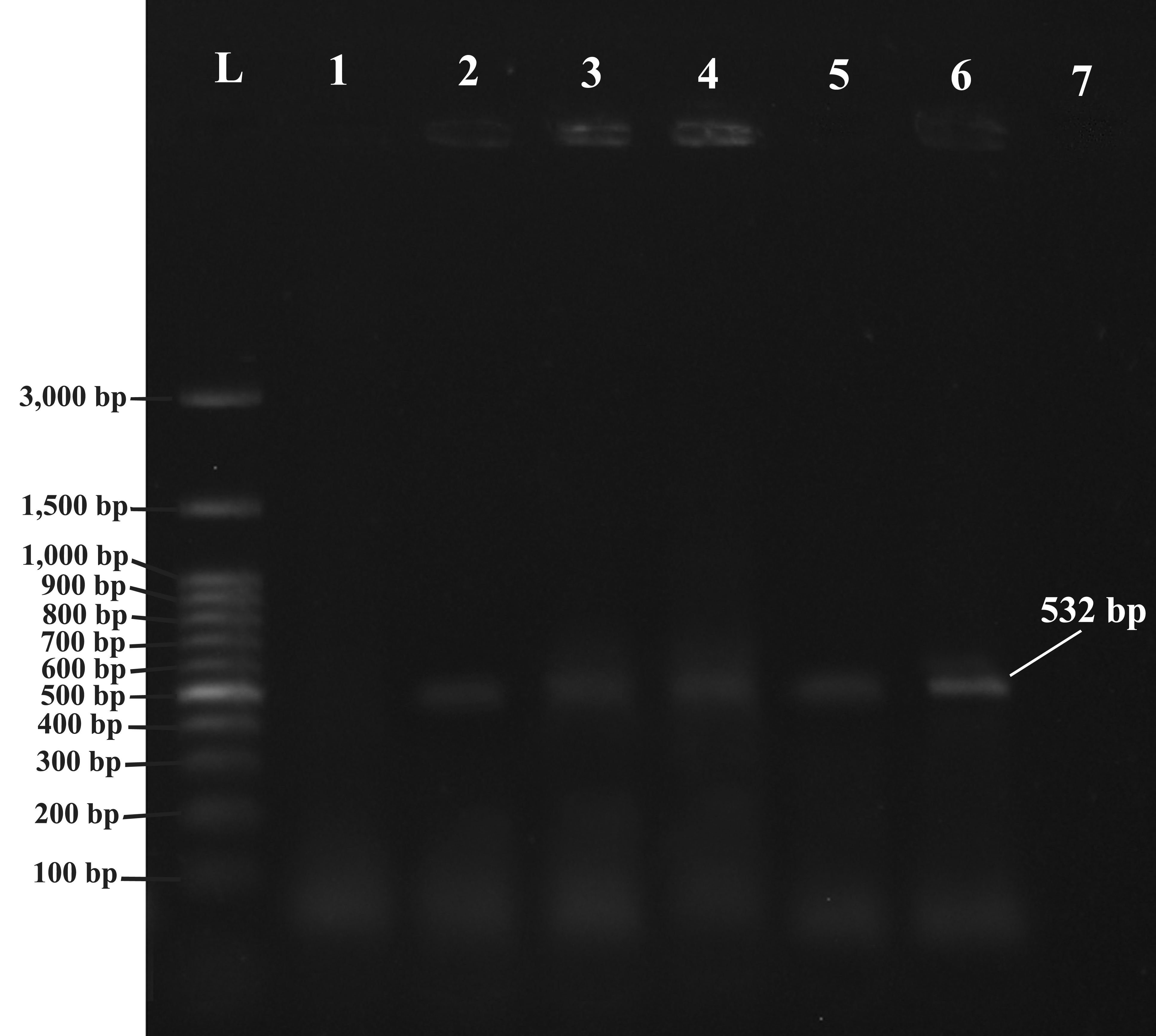

Supplement: Supplementary file 3 — Additional file 3: Figure S2. Agarose gel electrophoresis of PCR amplification products of mecA (532 bp) from Listeria monocytogenes isolates, L: Ladder (100 bp); Lanes 2, 3, 4 and 5: positive samples; Lane 6: positive control; Lane 7: negative control. [file 12866_2021_2326_MOESM3_ESM.jpg]

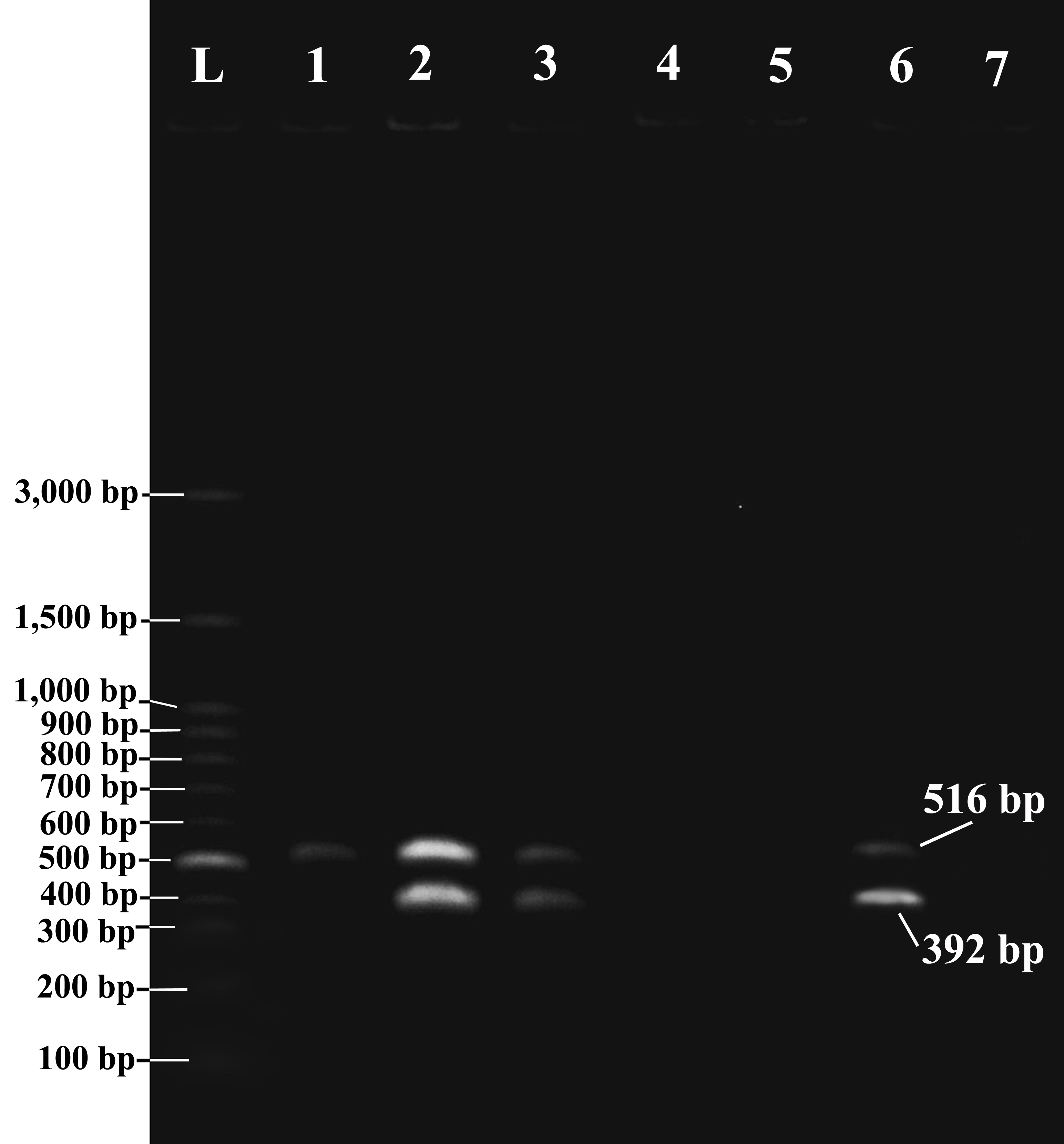

Supplement: Supplementary file 4 — Additional file 4: Figure S3. Agarose gel electrophoresis of PCR amplification products of blaSHV (392 bp) and blaTEM (516 bp) genes from the isolates of Salmonella spp., L: Ladder (100 bp); Lanes 1: positive samples for blaTEM, Lanes 2 and 3: positive samples for blaSHV blaTEM; Lane 6: positive control for blaSHV blaTEM; Lane 7: negative control. [file 12866_2021_2326_MOESM4_ESM.jpg]

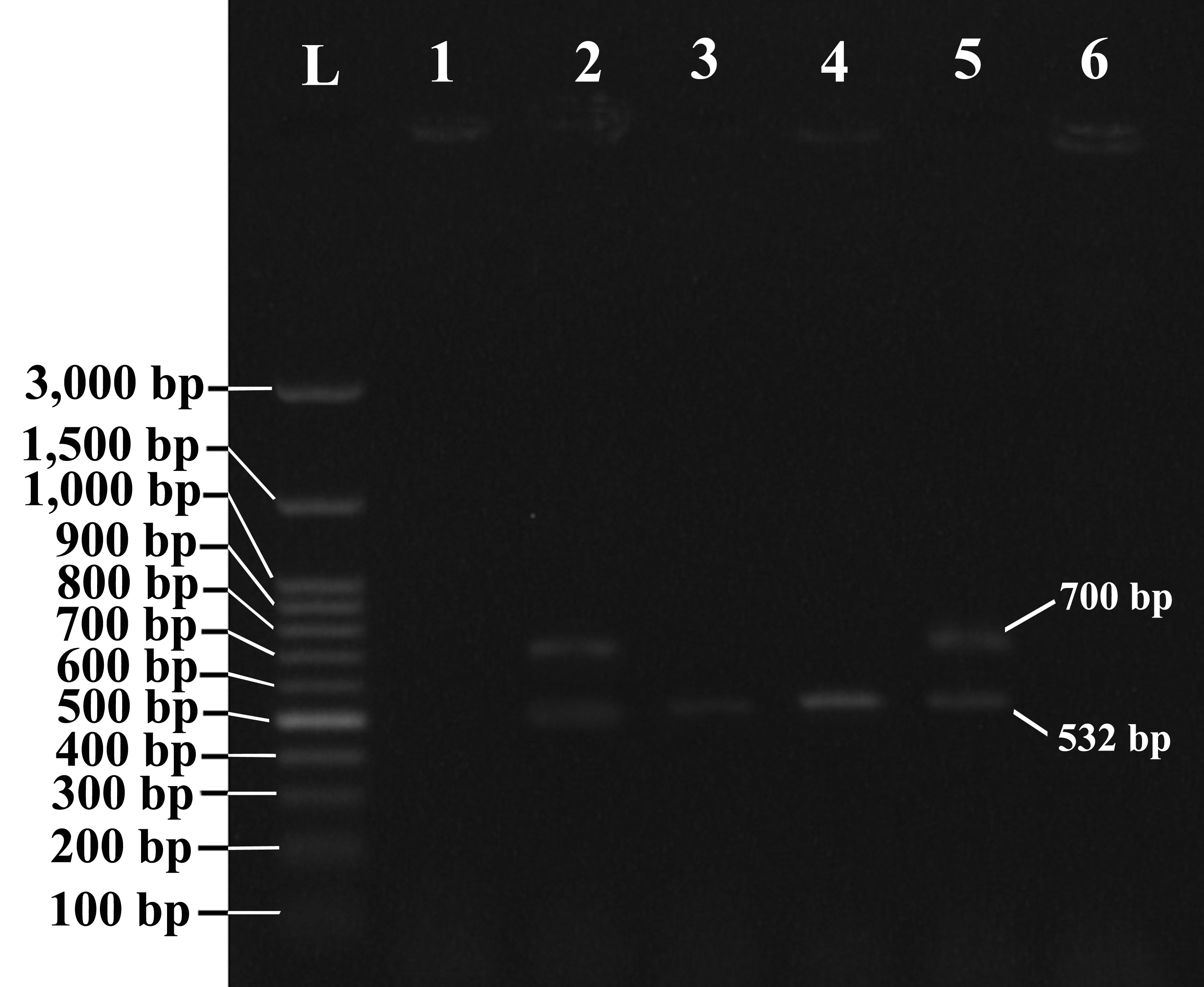

Supplement: Supplementary file 5 — Additional file 5: Figure S4. Agarose gel electrophoresis of PCR amplification products of mecA (532 bp) and blaZ (700 bp) from Staphylococcus aureus isolates, L: Ladder (100 bp); Lanes 2: positive sample for mecA and blaZ; Lanes 3 and 4: positive samples for mecA; Lanes 5: positive control for mecA and blaZ; Lane 6: negative control. [file 12866_2021_2326_MOESM5_ESM.jpg]
